# Neonatal Overnutrition Increases Testicular Size and Expression of Luteinizing Hormone β-Subunit in Peripubertal Male Rats

**DOI:** 10.3389/fendo.2018.00168

**Published:** 2018-04-13

**Authors:** Pilar Argente-Arizón, David Castro-González, Francisca Díaz, María J. Fernández-Gómez, Miguel A. Sánchez-Garrido, Manuel Tena-Sempere, Jesús Argente, Julie A. Chowen

**Affiliations:** ^1^Department of Endocrinology, Hospital Infantil Universitario Niño Jesús, Madrid, Spain; ^2^Instituto de Investigación La Princesa, Madrid, Spain; ^3^CIBER Fisiopatología de la Obesidad y Nutrición, Instituto de Salud Carlos III, Madrid, Spain; ^4^Department of Pediatrics, Universidad Autónoma de Madrid, Madrid, Spain; ^5^Department of Cell Biology, Physiology and Immunology, Instituto Maimónides de Investigacion Biomédicas de Córdoba (IMIBIC), Hospital Universitario Reina Sofia, University of Córdoba, Córdoba, Spain; ^6^IMDEA Food Institute, CEI UAM-CSIC, Madrid, Spain

**Keywords:** neonatal, overnutrition, leptin, hypothalamus, puberty

## Abstract

Proper nutrition is important for growth and development. Maturation of the reproductive axis and the timing of pubertal onset can be delayed when insufficient nutrition is available, or possibly advanced with nutritional abundance. The childhood obesity epidemic has been linked to a secular trend in advanced puberty in some populations. The increase in circulating leptin that occurs in association with obesity has been suggested to act as a signal that an adequate nutritional status exists for puberty to occur, allowing activation of central mechanisms. However, obesity-associated hyperleptinemia is linked to decreased leptin sensitivity, at least in adults. Here, we analyzed whether neonatal overnutrition modifies the response to an increase in leptin in peripubertal male rats, as previously demonstrated in females. Wistar rats were raised in litters of 4 (neonatal overnutrition) or 12 pups (controls) per dam. Leptin was administered sc (3 µg/g body weight) at postnatal day 35 and the rats killed 45 min or 2 h later. Postnatal overfeeding resulted in increased body weight and circulating leptin levels; however, we found no overweight-related changes in the mRNA levels of neuropeptides involved in metabolism or reproduction. In contrast, pituitary expression of luteinizing hormone (LH) beta-subunit was increased in overweight rats, as was testicular weight. There were no basal differences between L4 and L12 males or in their response to leptin administration in pSTAT3 levels in the hypothalamus at either 45 min or 2 h. In contrast, pJAK2 was found to be higher at 45 min in L4 compared to L12 males regardless of leptin treatment, while at 2 h it was higher in L4 leptin-treated males compared to L12 leptin-treated males, as well as L4 vehicle-treated rats. There were no changes in response to leptin administration in the expression of the neuropeptides analyzed. However, serum LH levels rose only in L4 males in response to leptin, but with no change in testosterone levels. In conclusion, the advancement in pubertal onset in males with neonatal overnutrition does not appear to be related to overt modifications in the central response to exogenous leptin during the peripubertal period.

## Introduction

Pubertal onset is the culmination of maturational processes that result in an increase in the activation of gonadotropin-releasing hormone (GnRH) neurons, which then activate the gonadotropin axis to induce maturation of the gonads and reproductive function (1–4). The timing of this phenomenon is determined by the interaction between an individual’s genetic makeup and environmental influences (5). For example, a suboptimal nutritional status, such as in anorexia nervosa or extreme physical exercise, has been shown to delay pubertal maturation in children (6, 7), and this has been interpreted as indicating that sufficient energy stores are required for reproductive functions to be capacitated, at least in females. In turn, it is very likely that the inverse is also true; that an abundance of available energy advances pubertal development. Indeed, studies suggest that the mean age of pubertal onset is advanced in the growing population of obese/overweight children in developed countries compared to their lean peers, with both boys and girls being affected (1, 2, 8–11). Although this trend in overall earlier pubertal development should not be confused with pathological precocious puberty, it could result in an increased risk for these individuals to develop hypertension or metabolic diseases in later life (12), emphasizing the importance in understanding the mechanisms underlying this phenomenon.

Circulating leptin levels are increased in obese children (13, 14), with the levels of this hormone reported to be inversely related to the age at menarche (15). Indeed, leptin is not only an important anorexigenic signal, modulating proopiomelanocortin (POMC) and neuropeptide Y/Agouti-related peptide (NPY/AgRP) neurons (16, 17), but it is also considered to be a permissive signal for pubertal onset (18–20) and an important link between metabolism and puberty (21). Patients or rodents that are genetically lacking leptin or that harbor LepR mutations lack pubertal development and are infertile (22–25), with leptin replacement normalizing the reproductive axis in leptin-deficient subjects (22, 23, 25). Although this hormone does not trigger pubertal onset, a minimum level of leptin levels/activity appears to be required for pubertal development and maintenance of the reproductive axis. Indeed, leptin treatment of normal mice has been shown to advance pubertal onset indicating that the levels of this hormone are important for pubertal timing (26).

The experimental model of neonatal overnutrition induced by a reduction in litter size results in advancement of the mean age of pubertal onset in both male and female rodents (27–29), which is most likely due, at least in part, to the rise in circulating leptin levels associated with their increased bodyweight/fat mass (28, 30). Moreover, we previously reported that during the peripubertal period, the central response to leptin is increased in neonatally overnourished female rats (31), suggesting that this increased leptin sensitivity could also participate in advancing the activation of the reproductive axis. However, it is unknown whether a similar phenomenon occurs in peripubertal male rats. Hence, the aim of the study presented here was to analyze whether early overnutrition affects hypothalamic leptin sensitivity in peripubertal male rats.

## Materials and Methods

### Animals

Wistar rats bred in the animal facilities of the University of Córdoba were employed. After mating and confirmation of pregnancy, dams were housed individually given free access to standard laboratory chow and tap water. On the day of birth [postnatal day (PND) 0], pups were arranged into two different litter sizes: 4 pups/litter (overnutrition; ON) and 12 pups/litter (control; Ct) with equal number of males and females in each. Cross-fostering was used. Only male offspring were analyzed in the studies reported here. Rats were kept under constant conditions of light (12 h of light, from 0730 h) and temperature (22–24°C). The experimental procedures were approved by the respective University Ethical Committee for Animal Experimentation (Royal Decree 53/2013) and conducted in accordance with the European Union guidelines for use of experimental animals (2010/63/EU).

### Experimental Design

On PND 22, the pups were weaned and housed in groups of four rats of the same experimental group per cage. All rats had free access to normal rat chow and water. Body weight (BW) gain and food intake were evaluated at PNDs 25, 30, and 35. As an external sign of puberty, balano-preputial separation was monitored between PNDs 30 and 35 (end of the study).

On PND 35 rats (*n* = 24/group) were injected sc with either rat leptin (3 µg/g BW; National Hormone and Pituitary Program, Torrance, CA, USA) or vehicle (saline + 0.1% BSA, 10 ml/kg), after a fasting period of 12 h. This dose of recombinant leptin was chosen as it activates leptin signaling cascades and modulates hypothalamic neuropeptide expression (31–33). Rats were injected with leptin and killed 45 min later for the analysis of intracellular leptin-signaling pathways in the hypothalamus and 2 h after hormone injection to study changes in gene expression in the hypothalamus and pituitary, in addition to intracellular leptin-signaling pathways. All rats were killed between 0900 and 1100.

Trunk blood was collected in tubes containing EDTA. Samples were centrifuged at 3,000 rpm (20 min) and plasma stored at −80°C until processed. After rapidly removing the brain, the hypothalamus was isolated on ice by making an anterior cut at the level of the optic chiasm, a posterior coronal section anterior to the mammillary bodies, two sagittal cuts parallel to the lateral ventricles and a dorsal horizontal cut at the level of the anterior commissure. The block was then immediately stored at −80°C until processed. The testes were removed and the surrounding fat removed before weighing and freezing. The pituitary gland and visceral (perigonadal) and subcutaneous (inguinal) fat pads were also removed, weighed, and stored at −80°C.

### Hormone Measurements

Plasma leptin and insulin levels were measured by multiplexed immunoassay following the manufacturer’s instructions (Millipore, Billerica, MA, USA). The sensitivity of the assay was 21.5 and 51 pg/ml for leptin and insulin, respectively. The intra-assay coefficient of variation (CV) was 8.2 and 7.3% and the inter-assay CV was 8.5 and 9.8% for leptin and insulin, respectively.

A radioimmunoassay supplied by the National Institutes of Health (National Hormone and Peptide Program, Torrance, CA, USA) was used to measure circulating luteinizing hormone (LH) concentrations. Rat LH-I-10 was labeled with ^125^I using iodo-gen tubes (Pierce Chemical Co., Rockford, IL, USA). Hormone concentrations are expressed using reference preparation LH-RP-3 as standard. The sensitivity of the assay was 3.75 ng/tube and intra-assay CV was less than 8%.

### Protein Extraction and Western Blotting

Hypothalami from rats sacrificed 45 min after acute leptin treatment were homogenized in lysis buffer [0.1 M NaH_2_PO_4_ (pH 7.4), 20% Triton X-100, 10% SDS, 0.5% NaN_3_, 0.5% sodium deoxycholate, 100 mM phenylmethylsulfonyl fluoride, and a cocktail of EDTA-free protease inhibitors (Roche Diagnostics, Mannhein, Germany)]. The lysates were centrifuged at 14,000 *g* for 10 min at 4°C, and the supernatants stored at −80°C until assayed.

Hypothalami from rats sacrificed 2 h after acute leptin treatment were processed according to RNeasy Plus Mini Kit protocol (QIAGEN Iberia), obtaining a protein fraction that was precipitated with acetone. The pellets were reconstituted in 3-[(3-cholamidopropyl) dimethylammonio] propanesulfonate CHAPS buffer (7 mM urea, 2 M thiourea, CHAPS 4% w/v, 0.5% v/v 1 M Tris pH 8.8). The protein concentration in each sample was determined by the method of Bradford (Bio-Rad Laboratories, Hercules, CA, USA) and 40 µg of protein were resolved on a 10% SDS-polyacrylamide gel under denaturing conditions, electro-transferred to polyvinyl difluoride membranes (Bio-Rad Laboratories), and the transfer efficiency determined by Ponceau red dyeing. Membranes were blocked in Tris-buffered saline (20 mM) containing 5% non-fat dried milk or BSA and 0.1% Tween 20 for 2 h, and incubated overnight at 4°C under agitation with the primary antibody at a concentration of 1:1,000. Primary antibodies toward the following proteins were used: anti-STAT3, phospho-STAT3 (Tyr705), phospho-Akt (Ser 473), phospho-ERK1/2, and phospho-JAK2 (Tyr1007/1008) from Cell Signaling (Boston, MA, USA), Akt, ERK1/2, and phospho-PTEN (Ser380/Thr383) from Santa Cruz Biotechnology (Santa Cruz, CA, USA), SOCS3 from Proteintech (Chicago, IL, USA), PTEN from Sigma (St. Louis, MO, USA), PTP1B from Novus Biologicals (Atlanta, GA, USA), and GAPDH from Anaspec (San Jose, CA, USA). Secondary antibodies conjugated with peroxidase were from Pierce Biotechnology (Rockford, IL, USA). Bound peroxidase activity was visualized by chemiluminescence (Bio-Rad Laboratories) and quantified by densitometry using an ImageQuant LAS 4000 mini system (GE Healthcare Little Chalfont, UK). All blots were normalized with Ponceau, GAPDH, or total protein to control for loading variability and all values were normalized to control values on each gel.

### RNA Preparation and Quantitative Real-Time PCR

Total RNA was extracted from hypothalami and pituitary glands by using RNeasy Plus Mini columns (QIAGEN Iberia). cDNA was synthesized from 2 µg of total RNA by using a high-capacity cDNA reverse transcription kit (Applied BioSystems). Relative mRNA levels in hypothalamic samples were assessed by quantitative real-time PCR and predesigned primers (Applied BioSystems) for AgRP (Rn01431703), GHRH (Rn00580832), GnRH (Rn00562754), Kiss1 (Rn00710914), Kiss1R (Rn00576940), LepR (Rn01433205), POMC (Rn00595020), somatostatin (Rn00561967), and NPY (Rn01410145). In the pituitary, the mRNA levels of the beta-subunit of LH (Rn00563443) and FSH (Rn01484594) were measured. Quantitative real-time PCR was performed by using assay on-demand kits (Applied Biosystems) and TaqMan universal PCR master mix (Applied Biosystems) according to the manufacturer’s protocol in an ABI PRISM 7000 sequence detection system (Applied Biosystems). Values were normalized to the housekeeping genes GAPDH (Rn99999916) and phosphoglycerate kinase 1 (Rn01474008). The ΔΔC_T_ method was used to determine relative expression levels, according to manufacturer’s guidelines. Statistics were performed using ΔΔC_T_ values (31).

### Statistical Analysis

ANOVA with repeated measures were used to analyze changes in weight and food intake over time. Results were analyzed using unpaired Student’s *t*-tests when only two groups were involved or two-way ANOVA for more than two groups. When indicated, a one-way ANOVA followed by a Scheffé *F*-test was then used (SPSS v.15.0, IBM Corp., Armonk, NY, USA). *P* < 0.05 was considered significant. Data are presented as the mean ± SEM.

## Results

### Body Weight, Length, Food Intake, and Fat Mass

At the onset of the study (birth), there were no differences between the two litter sizes in body weight (L4: 6.3 ± 0.1, L12: 6.1 ± 0.3 g) or length (L4: 5.0 ± 0.1, L12: 5.0 ± 0.1 cm).

**Figure 1:**
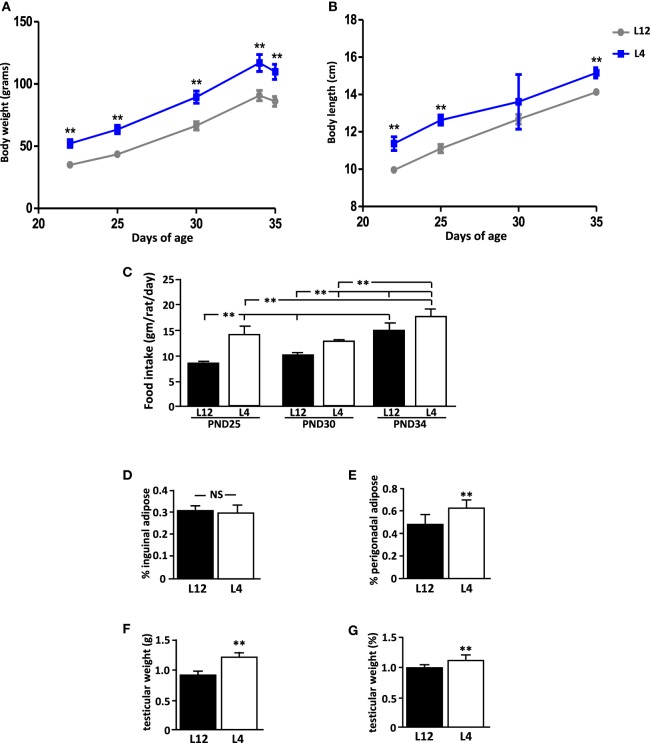
Effect of neonatal overnutrition (ON) on **(A)** body weight (BW), **(B)** body length (BL), **(C)** food intake, **(D)** inguinal adipose tissue, **(E)** perigonadal adipose tissue, **(F)** testicular weight, and **(G)** normalized testicular weight in peripubertal male rats raised in litters of 12 (L12) or litters of 4 pups (*n* = 12). PND, postnatal day. **p < 0.01. NS, non-significant. Results are presented as mean ± SEM.

When weaned at PND21, rats from the small litters weighed more than those from litters of 12 (L12) pups (*p* < 0.0001; Figure 1A). They were also longer (*p* < 0.0001; Figure 1B). These rats from small litters continued to weigh more and be longer than those from L12 pups throughout the study (Figures 1A,B, respectively), with the exception of body length at PND30 due to an unexplained variation in the weights of L4 pups. At the end of the study, L4 rats remained longer (*p* < 0.01) and weighed more (*p* < 0.01) than L12 rats, even after fasting before sacrifice.

Rats from small litters had a higher food intake (*p* < 0.01; Figure 1C) during the first part of the study (PND25 and PND30), but this difference was not significant at the end of the study (PND34). Food intake increased throughout the experiment in both groups.

At PND35, there was no difference between the experimental groups in the relative amount of inguinal adipose tissue (Figure 1D), while L4 rats had more perigonadal adipose tissue than L12 rats (*p* < 0.001; Figure 1E).

### External Signs of Puberty and Testicular Weight

No rat showed any signs of pubertal onset at any time throughout the study. At PND35, both the testicular weight (*p* < 0.01; Figure 1F) and relative testicular weight compared to BW (*p* < 0.01; Figure 1G) were greater in L4 rats than in L12 rats.

### Circulating Hormones during the Peripubertal Period in Response to Leptin

**Figure 2:**
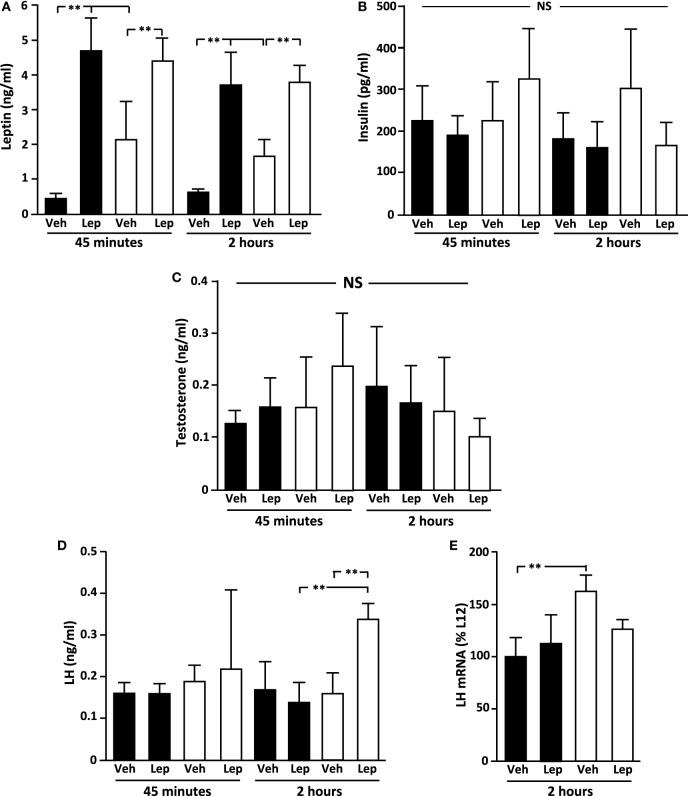
Serum **(A)** leptin, **(B)** insulin, **(C)** testosterone, and **(D)** luteinizing hormone (LH) levels in peripubertal male rats treated with vehicle (Veh) or leptin (Lep; 3 µg/g body weight) at 45 min and 2 h after administration and **(E)** LH mRNA levels in the pituitary 2 h after administration. Black bars: males raised in litters of 12 pups. White bars: rats raised in litters of 4 pups. **p < 0.01. NS, non-significant. *n* = 6/group. Results are presented as mean ± SEM.

Baseline serum leptin levels were higher in L4 rats compared to L12 rats (*p* < 0.001; Figure 2A) at both time-points. As expected, circulating leptin levels increased significantly (*p* < 0.001) after leptin treatment, with no difference between L4 and L12 rats in the levels of serum leptin after leptin injection (Figure 2A).

There was no effect of either litter size or leptin treatment on circulating insulin (Figure 2B) or testosterone (Figure 2C) levels. The basal concentration of LH was not different between groups. However, 2 h after leptin treatment, LH levels increased significantly in the overweight L4 rats resulting in higher levels compared to both L12 leptin-treated and L4 vehicle-treated rats (*p* < 0.01; Figure 2D).

### Pituitary Hormone Expression

The baseline levels of LH beta-subunit (LHβ) mRNA were higher in L4 rats compared to L12 rats (Figure 2E), with no significant effect of leptin, although there was no difference between the groups after leptin treatment. There were no differences between experimental groups in the mRNA levels of FSHβ, GH, ACTH (POMC), PRL, or TSHβ (Table 1).

**Table 1:** Relative levels of mRNA of selected genes in the pituitary of male peripubertal rats from litters of 12 (L12) or litters of 4 (L4) pups that were treated with vehicle (control, CT) or leptin (Lep) and sacrificed 2 h later.

	L12Ct	L12Lep	L4Ct	L4Lep	
FSHβ	100 ± 26.5	112.9 ± 17.9	117.9 ± 12.8	105.6 ± 22.5	NS
Prolactin	100 ± 11.9	88.6 ± 22.5	101.5 ± 25.5	97.8 ± 19.8	NS
GH	100 ± 23.7	110.1 ± 21.9	144.4 ± 41.8	83.6 ± 22.0	NS
TSHβ	100 ± 24.0	94.4 ± 11.3	87.4 ± 18.9	105.5 ± 15.8	NS
Proopiomelanocortin	100 ± 17.4	122.8 ± 24.1	95.4 ± 17.7	126.4 ± 26.7	NS

*n = 5–6/group. NS, not significant*.

### Expression of Neuropeptides and Receptors in the Hypothalamus

There was no effect of litter size or leptin treatment on the hypothalamic RNA levels of GnRH, Kiss, or KissR (Table 2). Likewise, there were no differences between experimental groups in the expression of the metabolic neuropeptides, NPY, AgRP, POMC, or orexin, as well as no difference in LepR (Table 2). The mRNA levels of the neuropeptides GHRH and somatostatin, involved in control of systemic growth and also metabolism (34), were unaffected by litter size or leptin treatment (Table 2).

**Table 2:** Relative levels of mRNA of selected genes in the hypothalamus of male peripubertal rats from litters of 12 (L12) or litters of 4 (L4) pups that were treated with vehicle (control, CT) or leptin (Lep) and sacrificed 2 h later.

	L12Ct	L12Lep	L4Ct	L4Lep	
NPY	100 ± 23.6	108.4 ± 24.7	99.7 ± 27.9	94.4 ± 13.7	NS
AgRP	100 ± 7.5	102.5 ± 29.3	116.3 ± 29.3	98.7 ± 20.2	NS
Proopiomelanocortin	100 ± 24.9	104.8 ± 7.7	124.1 ± 11.2	92.6 ± 14.0	NS
LepR	100 ± 7.9	112.6 ± 9.4	117.3 ± 12.3	111.9 ± 16.2	NS
Gonadotropin-releasing hormone	100 ± 4.6	80.0 ± 23.4	123.4 ± 23.2	91.2 ± 39.2	NS
Kiss	100 ± 17.7	117.4 ± 38.2	117.4 ± 23.8	106.2 ± 41.5	NS
KissR	100 ± 9.0	77.4 ± 25.2	97.3 ± 14.8	106.0 ± 29.1	NS
Somatostatin	100 ± 12.9	105.3 ± 8.8	92.9 ± 7.5	95.4 ± 8.1	NS
GHRH	100 ± 8.0	105.7 ± 6.1	88.6 ± 3.5	94.7 ± 7.3	NS

*n = 5–6/group. NS, not significant*.

### Intracellular Signaling

**Figure 3:**
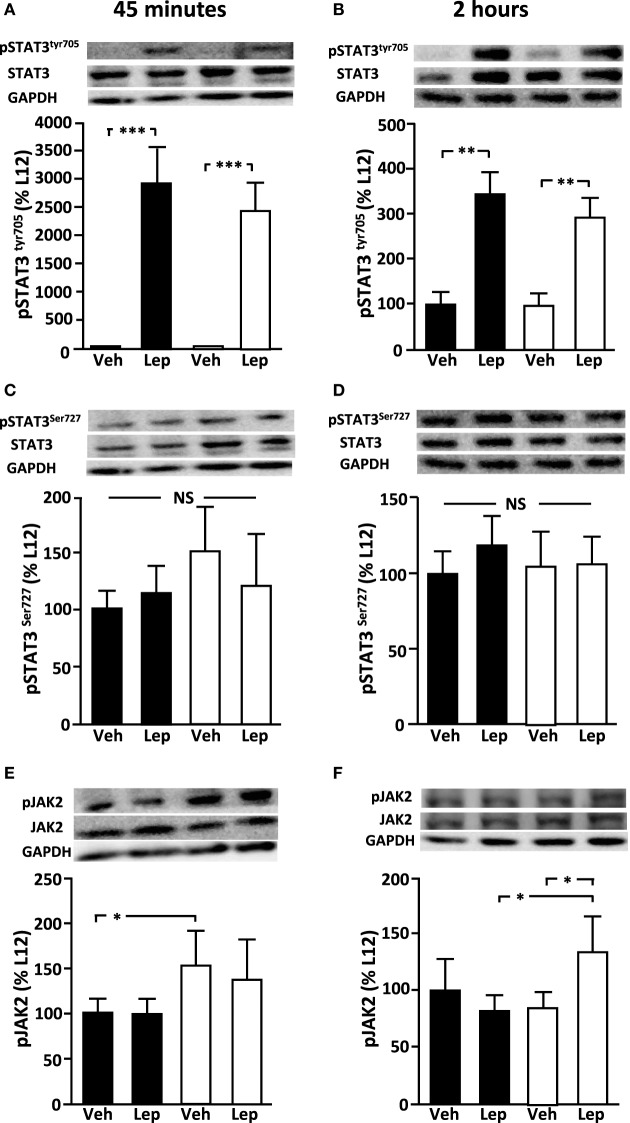
Relative levels of **(A,B)** pSTAT3^tyr705^, **(C,D)** pSTAT3^ser727^, and **(E,F)** pJAK2 in the hypothalamus of peripubertal male rats at 45 min (left panel) and 2 h (right panel) after either vehicle (Veh) or leptin (Lep; 3 µg/g body weight) administration. Representative western blots are shown for each protein. Black bars: males raised in litters of 12 pups. White bars: rats raised in litters of 4 pups. *p < 0.05; **p < 0.01. NS, non-significant (*n* = 6/group). Results are presented as mean ± SEM.

Basal levels of hypothalamic pSTAT3^Tyr705^ (Figures 3A,B) and pSTAT3^Ser727^ (Figures 3C,D) were not different between L12 and L4 rats at PND35. Leptin administration increased pSTAT3^Tyr705^ levels at both 45 min (*p* < 0.0001) and 2 h (*p* < 0.001). There was no difference in the response of L12 and L4 rats. In contrast, there was no effect of leptin treatment on pSTAT3^Ser727^ levels at either time-point. At 45 min, pJAK2 levels were higher in L4 compared to L12 rats in vehicle-treated rats (*p* < 0.05), with this difference not reaching significance in leptin-treated rats (Figure 3E). At 2 h, pJAK2 levels were higher in L4 leptin treated rats compared to L12 leptin-treated rats and L4 vehicle-treated rats (*p* < 0.05; Figure 3F).

There was no effect of litter size or leptin treatment on relative pAKT, pERK, SOCS3, pPTEN, or PTP1B levels at either 45 min or 2 h (Table 3).

**Table 3:** Relative protein levels of intracellular signaling molecules in the hypothalamus of male peripubertal rats from litters of 12 (L12) or litters of 4 (L4) pups that were treated with vehicle (control, CT) or leptin (Lep) and sacrificed 45 min or 2 h later.

	45 min					2 h				
	L12Ct	L12Lep	L4Ct	L4Lep		L12Ct	L12Lep	L4Ct	L4Lep	
pAKT	100 ± 12.3	86.6 ± 19.9	59.9 ± 22.8	85.4 ± 25.5	NS	100 ± 22.1	108.4 ± 20.6	109.1 ± 11.8	97.6 ± 5.5	NS
pERK1	100 ± 9.9	98.3 ± 10.6	105.3 ± 11.5	108.8 ± 19.8	NS	100 ± 45.2	112.3 ± 33.4	96.0 ± 35.7	70.5 ± 13.6	NS
pERK2	100 ± 3.6	112.6 ± 32.5	111.5 ± 31.1	99.5 ± 23.8	NS	100 ± 55.2	185.3 ± 65.4	142.0 ± 59.1	82.3 ± 38.1	NS
SOCS3	100 ± 18.3	105.1 ± 10.1	112.4 ± 9.8	106.0 ± 11.2	NS	100 ± 12.5	84.9 ± 7.4	101.9 ± 13.6	85.6 ± 8.7	NS
pPTEN	100 ± 5.3	108.9 ± 23.6	104.4 ± 5.9	102.5 ± 10.8	NS	100 ± 11.2	106.7 ± 9.5	102.8 ± 12.6	98.7 ± 14.6	NS
PTP1B	100 ± 7.2	83.5 ± 18.1	109.4 ± 7.3	101.0 ± 22.3	NS	100 ± 29.8	114.2 ± 43.6	113.0 ± 32.9	113.7 ± 34.0	NS

*n = 5–6/group. NS, not significant*.

## Discussion

Both over- and undernutrition during prepubertal life can affect the timing of pubertal onset in humans and rodents (1, 2, 5, 6, 8–10, 28), with changes in circulating leptin levels being one of the possible signals involved (15, 21). The experimental model used here to induce overnutrition from the time of birth, a reduction in litter size, has been repeatedly shown to increase body weight, fat mass, and length in male rats (28, 30–33, 35, 36) and has also been shown to advance the mean age of pubertal onset in both sexes (27–29).

Circulating leptin levels are increased as early as 10 days of life in both males and females with neonatal overnutrition (30) and here we found that they continued to be higher at PND35 in males with neonatal overnutrition, as previously reported for peripubertal (PND30) females (31). As leptin is an important permissive signal involved in the timing of pubertal onset (19, 23, 24, 37), the increase in this hormone during prepubertal life could be involved in the advancement of puberty in these animals. Obesity can be associated with leptin resistance (38, 39); however, we previously demonstrated that in female rats overweight due to neonatal overnutrition hypothalamic sensitivity to leptin is accentuated during the peripubertal period (31). In overweight peripubertal females (PND30), activation of hypothalamic pSTAT3 signaling in response to leptin, as well as the expression of LepR, were increased in females from small litters compared to those from large litters (31). In contrast, the increase in hypothalamic pSTAT in response to exogenous leptin did not differ between peripubertal male rats raised in small litters and controls, nor did the mRNA levels of LepR. There were no differences in the activation of MAP kinases, PTP1B, PTEN, or SOCS3 in response to neonatal overnutrition or leptin treatment in male rats, which is also in contrast to that found in females. These differences between males and females in the response to exogenous leptin could be due to innate differences between the sexes, but could also be in part due to the disparity in the levels of leptin reached after exogenous administration. Indeed, the levels obtained in females were much higher (31) than those observed in males. We are unable to explain this difference, as these treatments were performed by the same investigators during the same period of time as those of the female rats in our previous published studies, as were the determinations of leptin levels. It is possible that leptin is cleared from the circulation more rapidly in males compared to females, possibly by binding to specific receptors and being taken-up by target tissues or through renal clearance, but this remains to be demonstrated.

At 45 min, baseline hypothalamic pJAK2 levels were higher in L4 males compared to controls regardless of leptin treatment, which could reflect the higher baseline leptin levels. However, at 2 h after treatment pJAK2 levels were only higher in L4 males treated with leptin, as a result of the normalization of the levels in the L4 vehicle treated males. Thus, an increase in leptin sensitivity may not be involved in pubertal onset in male rats. However, it is also possible that modifications in leptin sensitivity have not yet occurred at PND35 and at an age closer to physiological pubertal onset differential leptin sensitivities might be found. What is clear is that the response of the intracellular pathways activated by leptin are not reduced in these overweight peripubertal males, suggesting that leptin resistance has not yet developed.

In peripubertal males, the baseline expression of metabolic neuropeptides was not affected by neonatal overnutrition or by leptin treatment. This is also in contrast to that previously reported in females (31). In peripubertal females, NPY mRNA levels were decreased and POMC mRNA levels increased in response to leptin in L4 overnourished rats, suggesting a possible increase in the central sensitivity to the anorexic actions of leptin. These changes could be related to the fact that by PND30 neonatally overnourished females have begun to normalize their body composition compared to controls (31) and at later ages females exposed to neonatal overnutrition are less apt to exhibit significant increases in body weight (30). In contrast, males are reported to be less capable of normalizing their food intake/body weight after neonatal overnutrition compared to females (30, 36).

Early overnutrition resulted in increased testicular mass, which might be associated with the advancement of pubertal onset previously reported in this experimental model (28). We might expect testosterone levels to increase with increasing testicular size, and although mean levels were not significantly different, it is possible that the secretory pattern of this steroid was affected. Indeed, testosterone values varied widely within each experimental group and this could be due to the occurrence of secretory bursts and troughs, possibly indicating pulsatile awakening and the nearing of pubertal onset. However, in rats, changes in testicular volume are not necessarily correlated with changes in serum testosterone levels in rodents (40, 41), as reported to occur in adult humans (42). In rodents, testicular volume increase from birth into early adulthood (43) and neonatal overnutrition appears to advance this age-related increase.

Basal circulating LH levels were not altered in these overnourished males, but basal LH β-subunit mRNA levels were increased in the anterior pituitary. Similar to the results regarding testosterone, single sampling of a hormone with pulsatile secretion could underlie the large standard errors in LH values. The higher levels of basal LH β-subunit expression in L4 rats could indicate greater synthesis of this gonadotrophin, with this gonadotrophin being stored in preparation for its release in response to stimulation. Indeed, LH secretion was significantly greater in response to leptin treatment in these overweight peripubertal pups, which could reflect higher LH stores available for release. It is also possible that in overweight L4 rats there is increased sensitivity to leptin to induce LH release, which could occur at the hypothalamic level (21, 44). However, at 2 h the mRNA levels of LH in L4 rats treated with leptin were no longer higher than those found in L12 rats. Leptin could possibly be directly inhibiting LH expression in these rats at the pituitary level, although recent studies suggest that this is not the case (45). It is also plausible that the increased release of LH itself could feedback to inhibit its own expression. In contrast, no effect of overnutrition or leptin was found on *GnRH* or *Kiss1* gene expression levels, which might indicate that a direct effect of leptin on the pituitary underlies these results.

As stated above, the peripubertal response of male rats to overnutrition and leptin administration was different from that reported previously in females (31). This could reflect the innate differences between the sexes in the neuroendocrine control of puberty and reproductive functions. Indeed, the GnRH/Kiss1 system is sexually dimorphic (46–48). Moreover, the interplay of neurons involved in metabolism and the interaction between nutrition and reproductive function also appears to be different between sexes. For example, the lack of leptin signaling in AgRP neurons was found to delay puberty in female mice, but with no significant effect in males (19). It is possible that the requirement of an adequate body weight for reproductive capacity is greater in females than in males, rendering them more sensitive to modifications in body weight during the peripubertal period. Indeed, we found few changes in the response of peripubertal males to leptin in the paradigm used here. It is possible that modifications in central leptin sensitivity are not involved in the advancement in pubertal onset of overnourished/overweight male rats or that central changes occur later than the age used here to analyze the response to leptin, i.e., PND35. However, the increase in testicular size and LH levels suggests that there are peripubertal differences in the reproductive system between control and overnourished male rats.

In conclusion, although pubertal onset can be modified by nutritional signals in both males and females, the sensitivity to these signals, as well as the mechanisms involved, may differ between the sexes. The advancement in the mean age at pubertal onset of both males and females as a result of early overnutrition/obesity is of concern considering the continuing rise in the prevalence of obesity in the pediatric population in developed countries and the possible long-term effects of this pubertal advance. Our results suggest that studies aimed at understanding the underlying mechanisms involved in this obesity-associated pubertal advancement should include both males and females.

## Ethics Statement

The authors have nothing to disclose.

## Author Contributions

PA-A: performed animal experiments, performed assays, and wrote manuscript; DC-G: performed animal experiments and performed assays; FD: performed assays; MF-G: performed assays; MS-G: performed animal experiments; MT-S: designed experiment and interpreted results; JA: designed experiment and interpreted results; JC: designed experiment, interpreted results, and wrote manuscript.

## Conflict of Interest Statement

The authors declare that the research was conducted in the absence of any commercial or financial relationships that could be construed as a potential conflict of interest.
